# Impact of Calcified Plaque Distribution on Symmetrical Stent Expansion After Intravascular Lithotripsy

**DOI:** 10.1016/j.jscai.2025.102616

**Published:** 2025-03-07

**Authors:** Takeshi Yamada, Norimasa Taniguchi, Tetsuya Hata, Shunsuke Nakajima, Shiori Kawakami, Akihiko Takahashi

**Affiliations:** a Cardiovascular Department, Sakurakai Takahashi Hospital, Kobe, Hyogo, Japan; b Kobe Women's University Graduate School, Kobe, Hyogo, Japan

**Keywords:** calcified lesion, intravascular lithotripsy, optical coherence tomography

## Introduction

The goal of stent implantation in percutaneous coronary intervention is to achieve a large stent lumen area with symmetrical, round-shaped expansion to minimize adverse events and improve clinical outcomes.^1^ Recently, intravascular lithotripsy (IVL) has emerged as a promising alternative to existing devices for treating calcified coronary arteries, modifying calcifications, and facilitating sufficient stent expansion.^2^ However, residual calcium plaques may still affect the subsequent stent expansion. This study aimed to investigate the impact of the baseline calcium angle and calcium fractures created by IVL on stent expansion with cross-sectional roundness in patients with calcified coronary lesions assessed using optical coherence tomography (OCT).

## Methods

This single-center retrospective study included 49 consecutive patients who underwent OCT-guided percutaneous coronary intervention with IVL from June 2022 to April 2024. Baseline and final OCT findings were obtained for all patients; images were taken immediately after IVL in 47 (94.9%) patients. OCT images were obtained for every 0.2 mm using a Dragonfly or Dragonfly OpStar catheter (Abbott Medical Japan) and were analyzed using Ultreon console version 2.0 (Abbott Medical Japan). In cases requiring rotational atherectomy, OCT images after atherectomy were assessed for baseline OCT findings. They were divided into 2 groups according to the maximum angle of calcium plaques in the maximum calcified segment among the target lesions observed on baseline OCT images: 180° to 270° and 271° to 360°. The lumen and vessel areas at baseline and after IVL, incidence of calcium fractures after IVL, stent area, and stent eccentricity index were evaluated.

## Results

The mean patient age was 75.7 ± 9.9 years. The maximum calcium angle at baseline OCT was 180° to 270° in 12 patients and 271° to 360° in 37 patients. Rotational atherectomy was performed in 5 (10.2%) patients prior to IVL catheter crossing, and the IVL catheter was successfully delivered in all patients; the mean IVL balloon diameter was 3.19 ± 0.39 mm, and a mean number of 82.1 ± 28.2 IVL pulses were delivered. Coronary stents were implanted in all patients after IVL; the mean stent diameter was 3.21 ± 0.41 mm. Postdilation of the stents was performed in 35 patients (71.4%). There were no significant differences in those patients' background and procedural characteristics between 2 groups according to baseline calcium angle using the χ^2^ test or the unpaired *t* test.

Assessment of the baseline OCT images indicated a mean reference diameter of 3.37 ± 0.53 mm (3.48 ± 0.53 in 180°-270° calcium group vs 3.33 ± 0.53 in 271°-360° calcium group; *P* = .395). The mean calcium angle, calcium length, and calcium thickness were 311° ± 59° (225° ± 35° vs 339° ± 31°; *P* < .001), 19.6 ± 9.4 mm (16.5 ± 7.5 mm vs 20.6 ± 9.8 mm; *P* = .199), and 1.05 ± 0.24 mm (0.93 ± 0.28 mm vs 1.09 ± 0.21 mm; *P* = .033), respectively. Calcified nodules were detected in 7 patients (16.7% vs 13.5%; *P* = .786). Calcium fractures were observed on OCT images after IVL in 40 (81.6%) patients and was more frequently observed in the group with circumferential calcium (45.5% vs 94.4%; *P* = .001).

**Figure 1:**
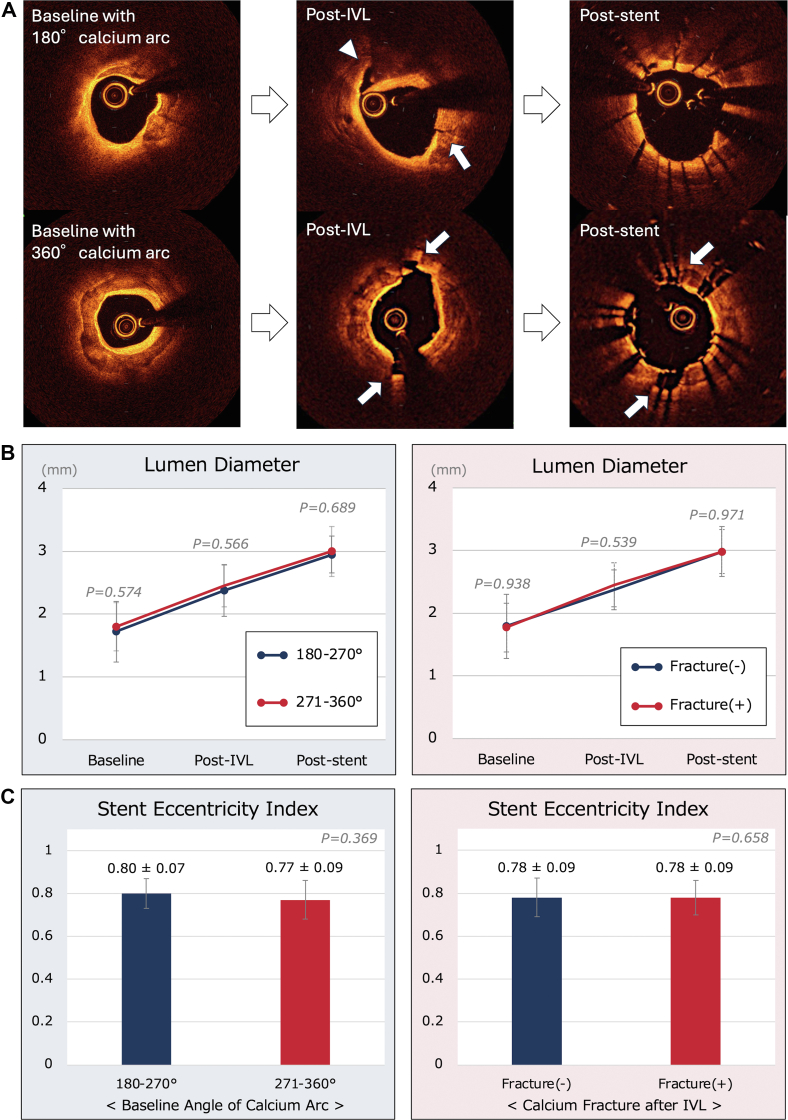
**Representative optical coherence tomography images, the changes in lumen diameter, and stent eccentricity index.** (**A**) Optical coherence tomography images. In the case of eccentric calcium, a small calcium fracture (white arrow) and medial dissection at the edge of sheet calcification (white arrowhead) were observed after intravascular lithotripsy (IVL). In the case of full circumferential calcium, 2 wide fractures (white arrow) were observed after IVL. Stents are well-expanded in both cases. (**B**) Lumen diameter in the maximum calcified segment. No significant differences were found between the groups according to the baseline calcium angle or optical coherence tomography–detected calcium fractures after IVL. (**C**) Stent eccentricity index in the maximum calcified segment. There were no significant differences between the groups according to the baseline calcium angle or calcium fractures after IVL.

On baseline OCT images of the maximum calcified segment, the mean lumen diameter was 1.78 ± 0.40 mm (1.72 ± 0.48 mm vs 1.80 ± 0.38 mm; *P* = .574), and the mean stent diameter on final OCT images was 2.99 ± 0.37 mm (2.95 ± 0.29 mm vs 3.00 ± 0.40 mm; *P* = .689). In the overall cohort, the mean stent area and mean stent eccentricity index were 7.18 ± 1.82 mm^2^ and 0.78 ± 0.09, respectively, with no significant difference between the 2 groups (Figure 1). Analysis of the impact of calcium fractures detected by OCT after IVL on stent expansion and stent eccentricity index (n = 47) revealed no significant difference (Figure 1). No complications related to the IVL procedure, such as coronary perforation and slow-flow/no-reflow phenomenon, were detected.

## Discussion

In this study, calcium fractures detected using OCT were more likely to be observed in the group with circumferential calcium than in the group without. However, the final stent expansion and eccentricity were not related to the calcium fractures detected using OCT after IVL. These findings may be attributable to the following: (1) IVL causes microfractures in calcium that are invisible with the resolution of OCT, and (2) approximately half of the major calcium fractures cannot be detected by OCT because of the presence of guidewire artifacts or the poor penetration depth of OCT, as reported in the micro–computed tomography study of cadaver heart.^3^ Thus, stents deployed after IVL are considered to have favorably expanded, regardless of OCT-detected calcium fractures.

## Conclusions

Coronary stents expanded favorably and concentrically without adverse events, irrespective of the baseline maximum calcium angle and the presence of OCT-detected calcium fractures after IVL.

## References

[1] Hamana T., Kawamori H., Toba T. (2023). Predictors of target lesion revascularisation after drug-eluting stent implantation for calcified nodules: an optical coherence tomography study. EuroIntervention.

[2] Kereiakes D.J., Di Mario C., Riley R.F. (2021). Intravascular lithotripsy for treatment of calcified coronary lesions: patient-level pooled analysis of the disrupt CAD studies. JACC Cardiovasc Interv.

[3] Kawai K., Sato Y., Hokama J.Y. (2023). Histology, OCT, and micro-CT evaluation of coronary calcification treated with intravascular lithotripsy: atherosclerotic cadaver study. JACC Cardiovasc Interv.

